# Exploring the possible relationship between ambient heat and sudden infant death with data from Vienna, Austria

**DOI:** 10.1371/journal.pone.0184312

**Published:** 2017-09-06

**Authors:** Thomas Waldhoer, Harald Heinzl

**Affiliations:** 1 Department of Epidemiology, Center for Public Health, Medical University of Vienna, Vienna, Austria; 2 Center for Medical Statistics, Informatics, and Intelligent Systems, Medical University of Vienna, Vienna, Austria; Helsingin Yliopisto, FINLAND

## Abstract

A non-linear relationship between maximum ambient temperature and number of sudden infant death syndrome (SIDS) cases had been reported for Montreal, Canada, for the warm season. In particular, high maximum ambient temperatures were found to be extra-hazardous for infants. The study was replicated with data from Vienna, Austria, applying the same statistical approach. Vienna is roughly comparable to Montreal with regard to temperatures in the warm season, size of population, and number of SIDS cases. Although the Viennese study was powerful enough to detect even smaller effects, the Montrealean results could not be confirmed. The Viennese results do not support the hypothesis of a strong effect of maximum ambient temperature on the risk of SIDS during the warm season.

## Introduction

The sudden infant death syndrome (SIDS) is a subcategory of the sudden and unexpected infant death (SUID) and accounts for 80% of such deaths [1]. SIDS is defined as “*the sudden death of an infant under one year of age*, *which remains unexplained after a thorough case investigation*, *including performance of a complete autopsy*, *examination of the death scene*, *and review of the clinical history*” [2, 3]. The thorough case investigation is crucial because SIDS is a diagnosis of exclusion. Although apparently occurring during sleep, the current SIDS definition does not refer to sleep [3].

Over the years, a plethora of potential risk and protective factors for SIDS has been suggested, studied and discussed. Based on these research efforts, various recommendations to reduce SIDS incidence have been developed [4–6]; thereby the *“back to sleep for every sleep”*-recommendation proved most useful in preventing SIDS (that is, infants should be placed for sleep in a supine position).

One class of potential risk factors can be subsumed under the term “overheating”. Among others, this may be due to overbundling, excessive bedclothes, head covering (thereby preventing heat loss through the face and head), and increased bedroom temperature (in particular in winter) [5, 7]. It is believed that the prone sleeping position, among others, prevents heat loss through the face [5, 7]. It has been suggested to label these conditions that threat the thermal regulation of the infant as thermal stress; thereby core body temperature—in distinction from hyperthermia—remains within normal limits [7].

Even though SIDS is usually less frequently observed in summer [3, 7], it seems natural to hypothesize that high outdoor temperatures may provide an additional source for SIDS-related thermal stress [8, 9]. Early studies, however, did not support this hypothesis. Using data from Taiwan from the period between 1981 and 1991, a study showed that SIDS risk decreased with increasing daily average temperature [10]. This result was confirmed with Taiwanese data from 1994 until 2003, thereby focusing on daily maximum instead of daily average temperature [11]. Similar results were reported for both the 1982–1983 and 1984–1985 North Carolina birth cohorts; SIDS risk was lower at higher daily maximum temperatures [12].

Data from four U.S. states (Arkansas, Georgia, Kansas, and Missouri) of the heat-wave summer of 1980 (May 1 through September 30, 1980) showed no relationship between SIDS risk and both mean and maximum daily temperature [13].

Recently, a roughly U-shaped association between ambient temperature and number of SIDS cases had been reported for the city of Montreal, Quebec, Canada, during April through October [8]. The authors considered all SIDS cases up to one year of age for the years 1981–2010 [8]. Using a case-crossover design, they found a trough of around 20°C in maximum outdoor temperature at SIDS day and an odds ratio greater than 3 when comparing SIDS risk at 30°C to 20°C, respectively. We investigated whether the reported association between maximum outdoor temperature and SIDS risk could be verified in Vienna, Austria, a European city roughly comparable to Montreal with regard to temperatures in the warm season, size of population, and number of SIDS cases.

## Materials and methods

In order to independently replicate the Montrealean results with SIDS data of Vienna, the original study design was emulated as close as possible.

*Statistics Austria*, the statistical office of Austria, provided anonymized data of Viennese SIDS cases up to one year of age for the years 1984–2014; SIDS cases with mention of autopsy were included in the analyses (ICD10 R95.0, ICD9 798.0 with mention of autopsy). As *Statistics Austria* routinely collects SIDS data and provides them in anonymized form for scientific research, no formal vote of an ethics committee was required for this study.

Meteorological measurements were provided by the *Zentralanstalt fuer Meteorologie und Geodynamik*, the meteorological office of Austria. From 1984 to 2014, the number of weather monitoring stations within the municipal area of Vienna increased from three to seven; their measurements were averaged before further use.

For the case-crossover design either the measurements at a SIDS or control day or the day before a SIDS or control day were considered. Control days for a SIDS case are composed of the remaining same weekdays of the month where the SIDS death occurred. The case-crossover design led to a conditional logistic regression model which allowed to assess the association between maximum outdoor temperature and SIDS risk, adjusted for mean relative humidity. Maximum outdoor temperature was flexibly modelled with natural cubic splines with knots placed at the 10th, 50th, and 90th percentiles [14, 15]. According to Auger et al. (2015), analyses were performed for all ages of the SIDS cases combined (0–364 days) and for the early and late postneonatal periods, that is, 1–2 months (28–89 days) and 3–11 months (90–364 days). For the neonatal period (0–27 days), the number of SIDS cases was too small. Additionally, analogous sensitivity analyses as in Auger et al. (2015) were performed.

A two-sided significance level *α* = 0.05 was applied; and a 95% confidence interval (CI) was reported for each odds ratio. All statistical calculations were performed with SAS version 9.4 (SAS Institute Inc., Cary, NC, USA).

## Results

A total of 187 SIDS cases with mention of autopsy were identified; they subdivide into 17, 74 and 96 cases between the age-groups neonatal, 1–2 months and 3–11 months, respectively. The pooled standard deviations of the maximum daily temperature within matched case-control sets were 4.54°C and 4.68°C for the day before and the day of a SIDS event, respectively.

Monthly values of mean maximum temperature and mean relative humidity at case and control days for the day before and the day of a SIDS event are shown in Table 1.

**Table 1:** Monthly weather conditions of SIDS case and control days, Vienna, April–October 1984–2014.

		Mean maximum temperature [°C (range)]				Mean relative humidity (%)			
	No. of SIDS	Previous day		Same day		Previous day		Same day	
Month		Cases	Controls	Cases	Controls	Cases	Cont.	Cases	Cont.
April	31	15.2 (7.4, 25.2)	16.5 (6.4, 27.0)	14.4 (5.3, 24.5)	16.2 (2.8, 27.4)	76.6	77.4	82.2	79.5
May	30	21.1 (10.8, 28.1)	20.9 (9.3, 29.3)	20.2 (9.3, 26.6)	20.9 (8.2, 31.5)	81.3	76.0	80.7	77.6
June	22	23.3 (17.1, 35.1)	22.5 (14.7, 34.6)	24.1 (15.9, 30.6)	22.9 (13.0, 33.4)	77.6	79.1	75.9	78.5
July	18	28.9 (23.0, 34.0)	26.7 (18.1, 33.8)	27.9 (19.5, 34.1)	25.7 (18.3, 34.2)	75.5	77.6	75.7	78.5
August	27	26.3 (18.5, 33.0)	26.2 (13.5, 35.2)	26.0 (18.8, 37.0)	26.4 (14.6, 36.4)	80.0	82.2	77.1	80.2
September	16	20.2 (13.1, 27.4)	20.3 (13.0, 30.9)	20.9 (11.3, 27.7)	20.6 (13.7, 28.5)	85.0	84.0	85.5	84.0
October	43	15.3 (5.9, 26.8)	14.6 (2.8, 23.5)	15.0 (5.6, 27.7)	14.5 (2.8, 23.5)	88.8	89.6	88.7	89.8

**Fig 1:**
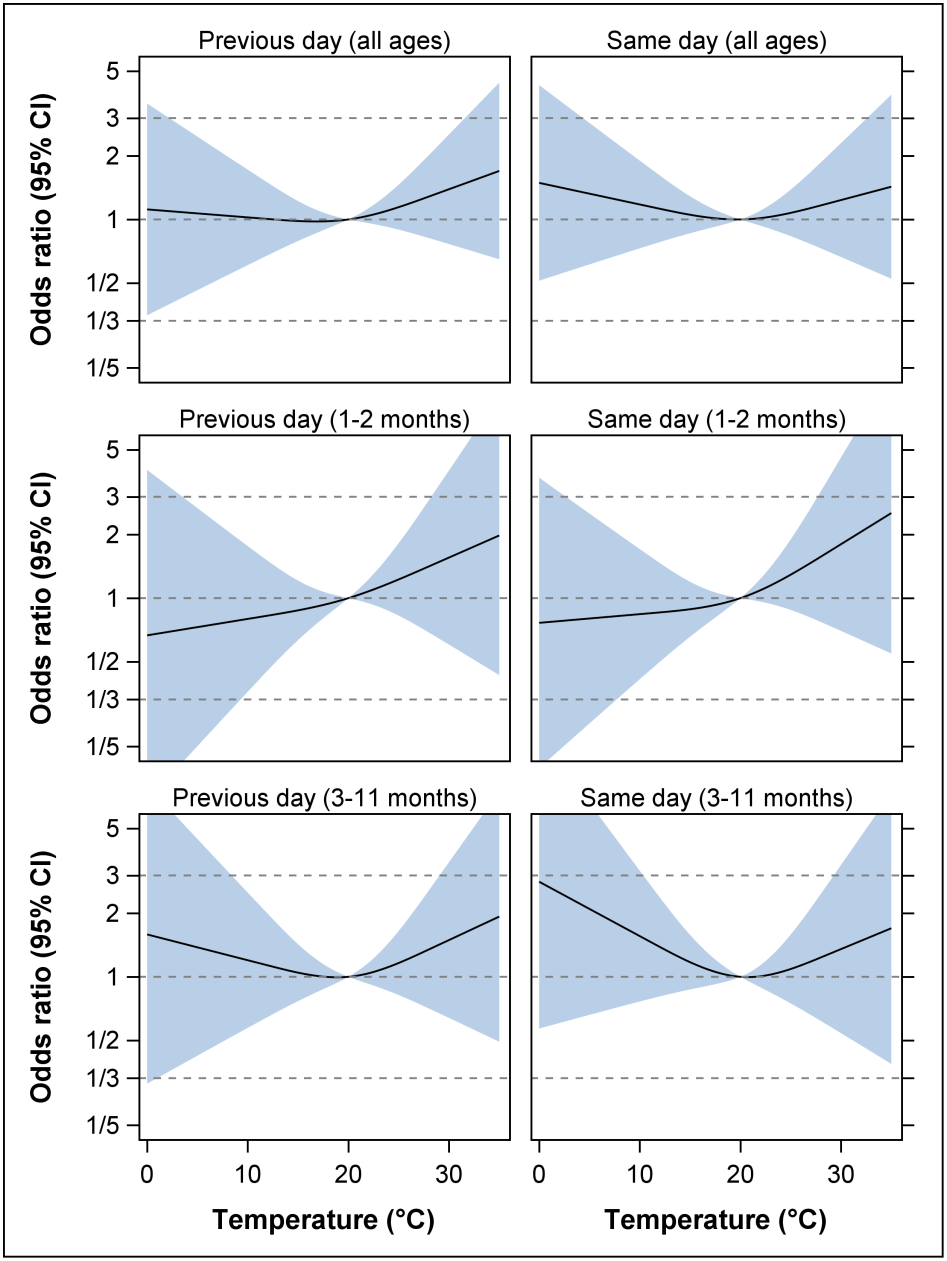
Association between maximum outdoor temperature and SIDS cases with mention of autopsy, Vienna, April–October 1984–2014. For the day before and the day of the SIDS event, odds ratios (solid black line) and 95% CI’s (transparent blue area) are shown for all ages (0–364 days) and two postneonatal periods. All odds ratios are relative to the reference temperature of 20°C, and are adjusted for mean relative humidity. The odds ratios are presented on the logarithmic scale.

No statistically significant associations between maximum daily temperature and SIDS could be found in the spline models (Fig 1); neither for all ages combined, nor for the two postneonatal periods; neither for the day before, nor for the day of the SIDS event, respectively. For all ages combined, the odds-ratios for a SIDS case at previous day temperatures of 24°C, 27°C and 30°C compared to 20°C were 1.10 (95% CI: 0.92, 1.33), 1.23 (95% CI: 0.84, 1.80), and 1.39 (95% CI: 0.77, 2.52), respectively. At same day temperatures, these odds-ratios were 1.05 (95% CI: 0.87, 1.27), 1.13 (95% CI: 0.76, 1.68), and 1.23 (95% CI: 0.67, 2.29), respectively.

Additional sensitivity analyses also did not reveal any significant effect of maximum daily temperature on SIDS (see S1 File). These sensitivity analyses involved changes in location and quantity of spline knots, exclusion of relative humidity from models, considering summer months (June–August) or SIDS cases aged 3–6 months (90–212 days) only, using the temperature at the second day before SIDS death, including 14 Viennese SIDS cases without mention of autopsy, and including further cities scattered all over Austria and thereby increasing the sample size to 276 SIDS cases with mention of autopsy (note that Vienna is by far the largest Austrian city).

It is interesting to ask for effects sizes that can be detected with such a study [16]. When assuming 180 matched case-control sets with 3 controls per set, a pooled standard deviation of the maximum daily temperature within matched case-control sets of 4.5°C, and a two-sided *α* = 0.05, the corresponding study will have 56%, 83%, 95% and 99.8% power to detect an odds-ratio of 1.5, 1.75, 2.0 and 2.5 for a SIDS case at a temperature of 30°C compared to 20°C, respectively.

## Discussion

Recently, Auger et al. (2015) described clear effects of ambient temperature on the number of SIDS cases in Montreal in the warm season. In spite of the same study design and statistical analysis approach we could not find any significant associations in a Viennese data set though numbers of SIDS cases as well as urban environment are similar in both cities. A comparison of the present Table 1 with Table 2 of Auger et al. (2015) confirms that maximum temperatures in Vienna and Montreal are quite similar during the warm season; relative humidity is higher in Vienna.

Considering all ages combined and same day temperatures, Auger et al. (2015) reported an estimated odds ratio of 3.18 when comparing 30°C to 20°C. Such a large effect is not covered by the corresponding odds ratio confidence intervals in the Viennese study; neither for the previous (95% CI: 0.77, 2.52) nor for the same day temperature (95% CI: 0.67, 2.29). The above sample size calculations suggest that the statistical power of the Viennese study is virtually 100% for detecting such an odds ratio.

At the same time, our results do not completely contradict those of Auger et al. (2015). Small increases in SIDS risk are observed for increasing maximum temperatures throughout; and even a flattened U-shaped relationship with a trough of around 20°C is observed in four out of six scenarios (Fig 1). This impression is to some extent supported by the sensitivity analyses (see S1 File). But then again, course and width of the pointwise confidence bands provide a clear warning against any over-interpretation of the non-significant effect estimates; even a negative relationship between SIDS risk and maximum temperature is still feasible.

In conclusion, the results of the present study do not support the hypothesis of a strong effect of maximum ambient temperature on the risk of SIDS during the warm season, although smaller effects cannot be excluded.

How can the gap between the Montrealean and the Viennese results be explained? It is self-evident, that the occurrence of a statistical error, either Type I (false positive) or Type II (false negative), respectively, cannot be ruled out. Moreover, replication studies generally tend to show smaller effects in absolute value than the studies they refer to. This has among others been observed for the North Carolina birth cohorts where the statistical model with the greatest effect in the 1982–1983 cohort was chosen as the final model which thereafter showed a smaller effect in the 1984–1985 replication cohort [12].

Although not solely focusing on the warm season, the North Carolina study together with two Taiwanese studies brings up another issue for consideration: these studies report lower SIDS risks for higher temperatures [10–12]. Additionally, there is the heat-wave study from four U.S. states that showed no relationship between SIDS risk and outdoor temperature between May and September 1980 [13]. Auger et al. (2015) referred to the results of Chang et al. (2013) and proposed that “*Taiwan has a mild climate with a population accustomed to heat*, *which may mitigate the impact of temperature on SIDS*” [8, 11]; however, Mage (2015) did not endorse this proposal [17].

We consider two reasons most crucial for explaining all these contradicting results: the SIDS definition and the infant’s actually experienced temperature.

Remember, SIDS is a diagnosis of exclusion [2, 3]. Now, Beckwith (2003) criticized that “*current definitional criteria [*…*] leave pathologists free to apply this designation either too liberally or too restrictively*” [3]; Kinney and Thach (2009) stated that “*no single definition of SIDS is universally accepted*, *and contradictions among SIDS studies are due in part to the use of various definitions of the syndrome around the world*” [1]; and Byard et al. (2007) warned that “*merely because a definition has been cited in a paper does not mean that all of the cases analysed have been diagnosed according to the cited criteria*” [18]. These concerns are supported by a study of Austrian infant mortality; the spatial distribution of the standard mortality ratios for SIDS was inversely correlated with nearly all remaining causes of infant mortality (infections and respiratory diseases, peripartal problems, immaturity, malformations, all other) [19].

Furthermore, outdoor temperature measurements may not be a good substitute for the actually experienced temperature by the individual infant. Since the use of air conditioning systems in private households is more frequent in Quebec (42% in 2009) than in Vienna (1.8% in 2012), indoor temperature in Montreal may differ from outdoor temperature to a greater extent than in Vienna [20, 21]. On the one hand, this argument may sound counterintuitive as it implies an even weaker effect of outdoor ambient temperature on SIDS mortality in Montreal than in Vienna due to lower indoor temperatures; on the other hand, however, children bedded in air-conditioned rooms may be too warmly dressed because of suspected low indoor temperature and air draft; consequently, they may be prone to overheating due to overbundling, excessive bedclothes or head covering [5, 7].

The issue of the actually experienced temperature has already been brought forward by De-Kun Li (senior research scientist at the Kaiser Permanente Northern California Division of Research) when commenting on the study of Auger et al. (2015) [22]. It constitutes a serious limitation of all studies (including the present one) that consider outdoor temperature when studying SIDS risk.

## Supporting information

S1 File Additional sensitivity analyses. (PDF)
