# Supplementary material for: Exploring the possible relationship between ambient heat and sudden infant death with data from Vienna, Austria
Source: PLoS One. 2017 Sep 6;12(9):e0184312. doi: 10.1371/journal.pone.0184312 (PMC5587259; doi:10.1371/journal.pone.0184312)
Supplement: S1 File — (PDF) [file pone.0184312.s001.pdf]

Additional sensitivity analyses for the PLOS ONE article “Exploring the possible relationship between ambient heat and sudden infant death with data from Vienna, Austria”

by Thomas Waldhoer and Harald Heinzl, 2017

Scenario 1 (corresponds to Figure 1 of the PLOS ONE article): Association between maximum outdoor temperature and SIDS cases with mention of autopsy, Vienna, April–October 1984–2014. Maximum temperature was flexibly modelled with natural cubic splines with knots placed at the 10th, 50th, and 90th percentiles. For the day before and the day of the SIDS event, odds ratios (solid blue line) and 95% CI’s (transparent blue area) are shown for all ages (0-364 days) and two postneonatal periods. All odds ratios are relative to the reference temperature of 20°C, and are adjusted for mean relative humidity. The odds ratios are presented both on the original and the logarithmic scale.

P-Value for spline-modelled effect of maximum temperature:

	# of SIDS cases	Previous day	Same day
all ages (0-364 days)	187	0.56	0.67
1-2 months (28-89 days)	74	0.52	0.40
3-11 months (90-364 days)	96	0.61	0.42

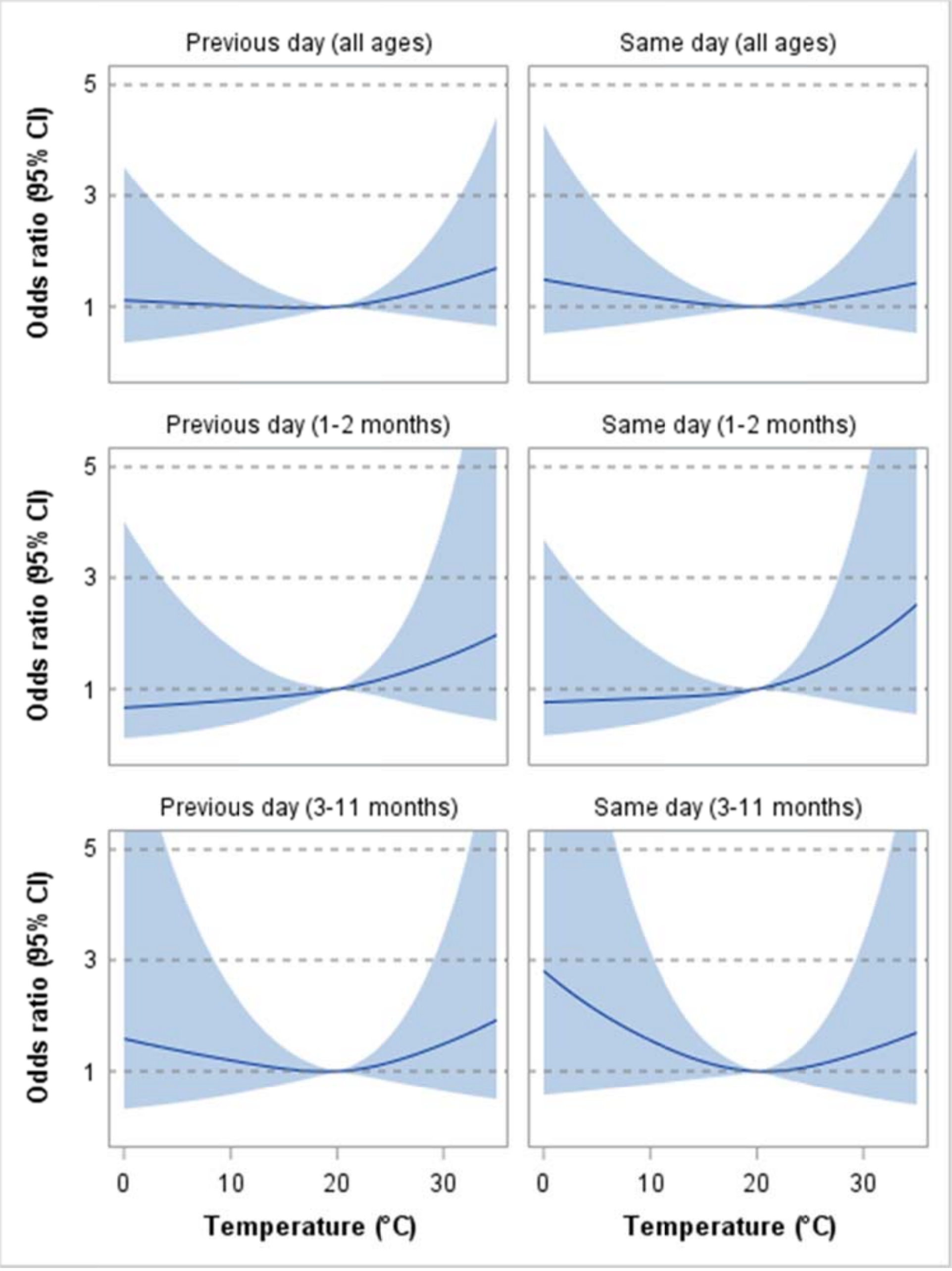

Previous day (all ages)
Same day (all ages)
Previous day (1-2 months)
Same day (1-2 months)
Previous day (3-11 months)
Same day (3-11 months)
Odds ratio (95% CI)
5
3
1
0
10
20
30
Temperature (°C)

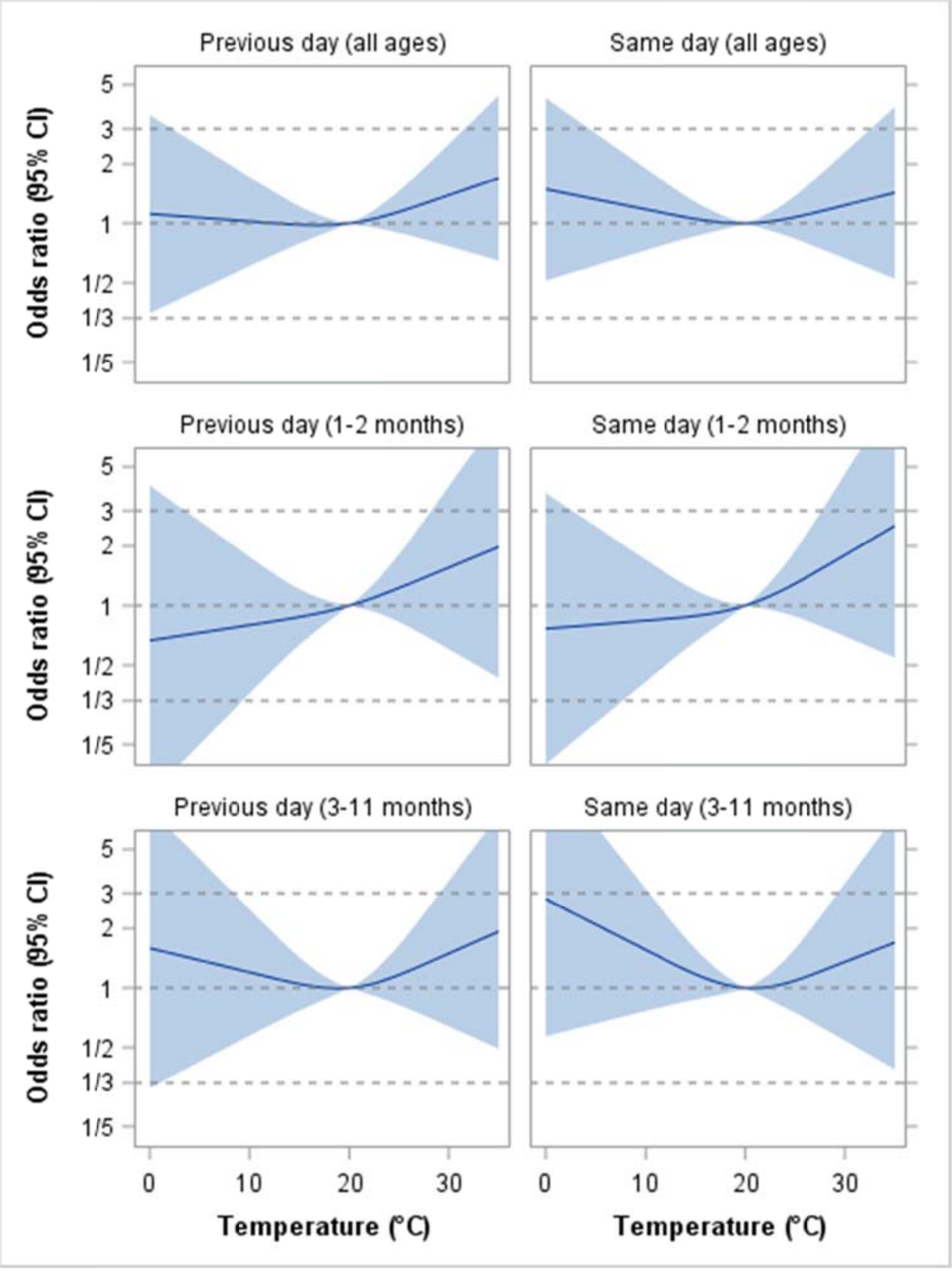

Previous day (all ages)
Same day (all ages)
Previous day (1-2 months)
Same day (1-2 months)
Previous day (3-11 months)
Same day (3-11 months)
Odds ratio (95% CI)
5
3
2
1
1/2
1/3
1/5
0
10
20
30
Temperature (°C)

Scenario 2: Association between maximum outdoor temperature and SIDS cases with mention of autopsy, Vienna, April–October 1984–2014. Maximum temperature was flexibly modelled with natural cubic splines with knots placed at the **5th, 50th, and 95th percentiles**. For the day before and the day of the SIDS event, odds ratios (solid blue line) and 95% CI's (transparent blue area) are shown for all ages (0-364 days) and two postneonatal periods. All odds ratios are relative to the reference temperature of 20°C, and are adjusted for mean relative humidity. The odds ratios are presented both on the original and the logarithmic scale.

P-Value for spline-modelled effect of maximum temperature:

	# of SIDS cases	Previous day	Same day
all ages (0-364 days)	187	0.59	0.73
1-2 months (28-89 days)	74	0.53	0.42
3-11 months (90-364 days)	96	0.63	0.46

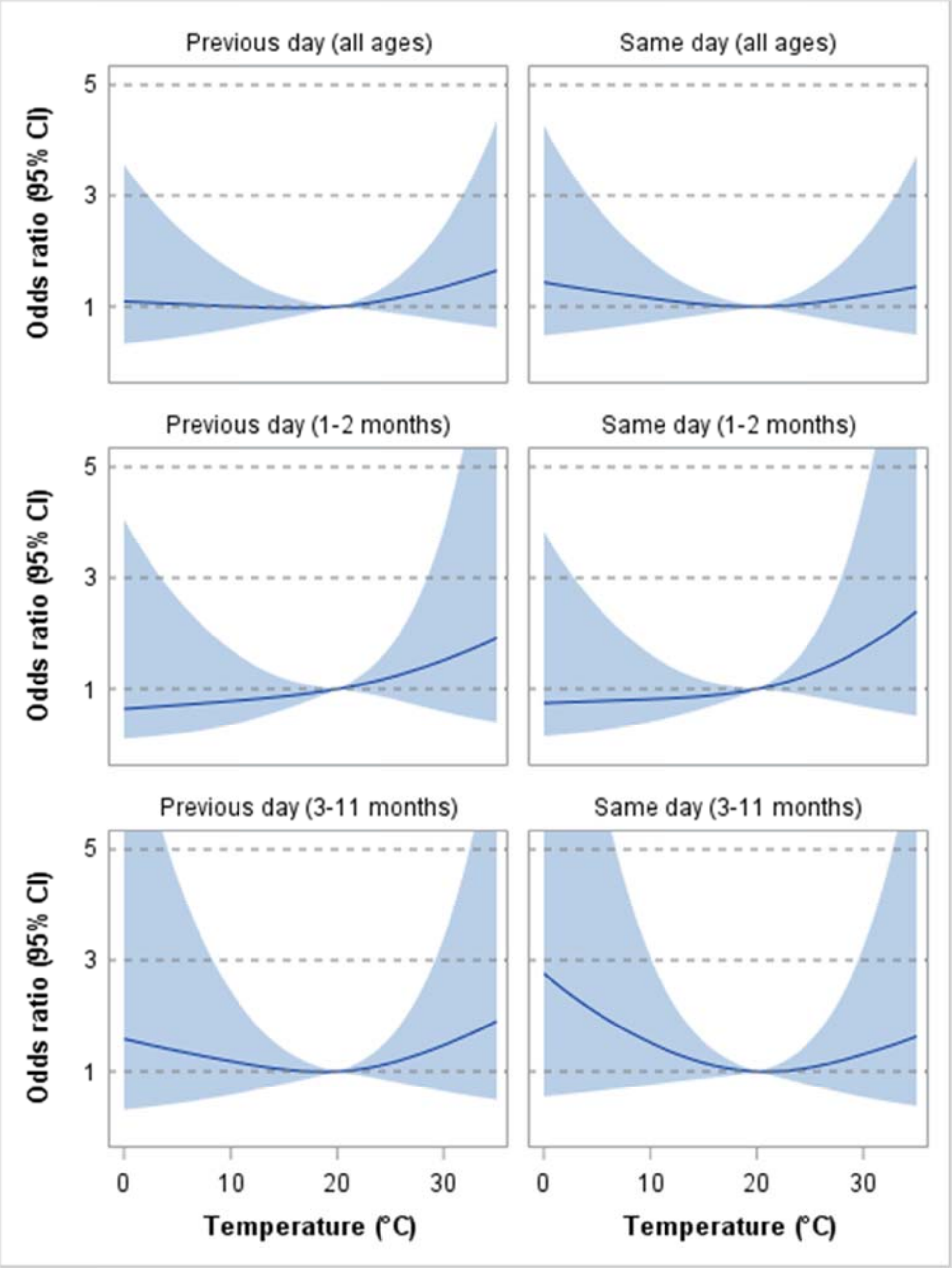
Previous day (all ages)
Same day (all ages)
Previous day (1-2 months)
Same day (1-2 months)
Previous day (3-11 months)
Same day (3-11 months)
Odds ratio (95% CI)
5
3
1
0
10
20
30
Temperature (°C)

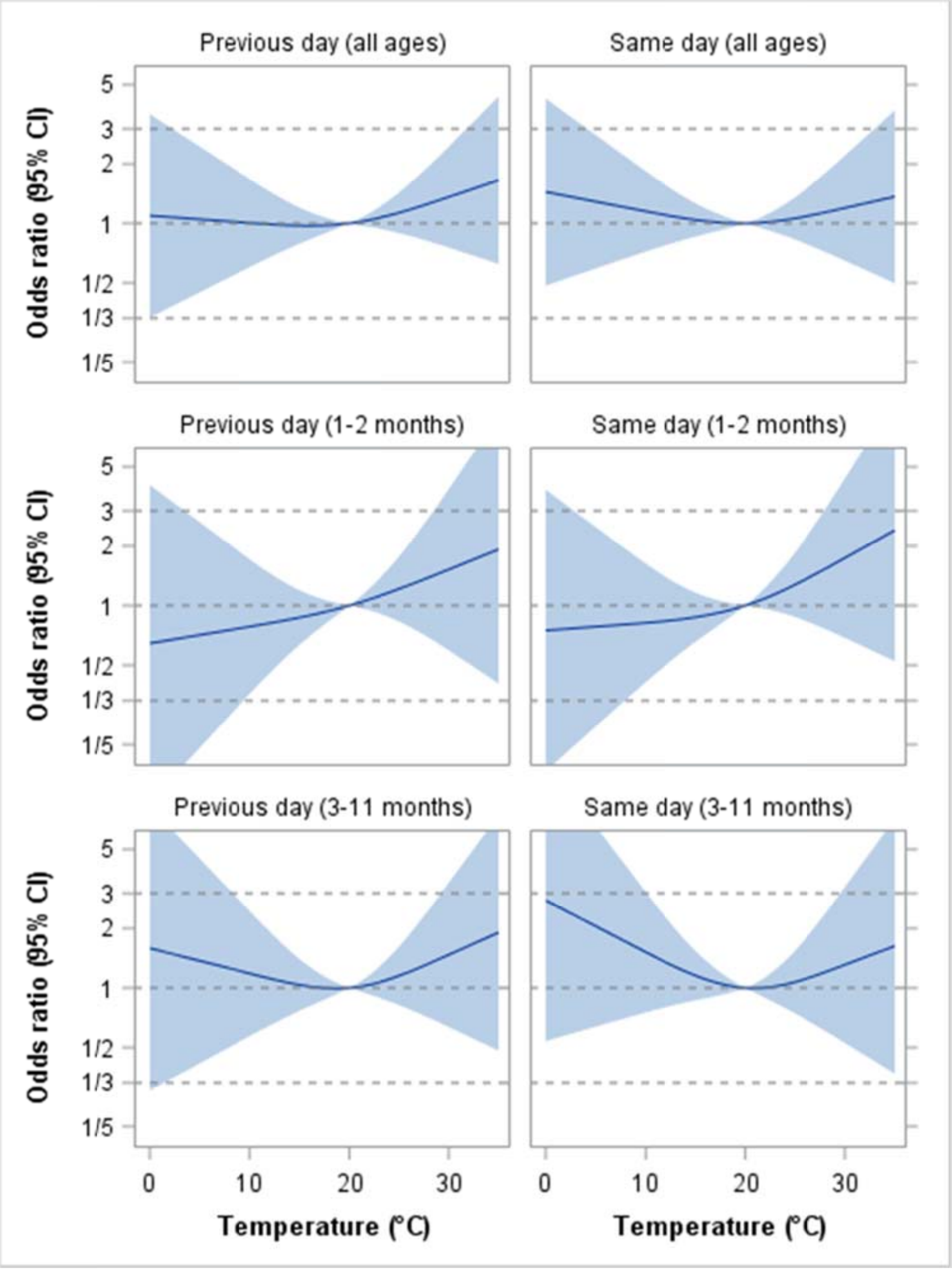
Previous day (all ages)
Same day (all ages)
Previous day (1-2 months)
Same day (1-2 months)
Previous day (3-11 months)
Same day (3-11 months)
Odds ratio (95% CI)
5
3
2
1
1/2
1/3
1/5
0
10
20
30
Temperature (°C)

Scenario 3: Association between maximum outdoor temperature and SIDS cases with mention of autopsy, Vienna, April–October 1984–2014. Maximum temperature was flexibly modelled with natural cubic splines with knots placed at the **5th, 35th, 65th, and 95th percentiles**. For the day before and the day of the SIDS event, odds ratios (solid blue line) and 95% CI's (transparent blue area) are shown for all ages (0-364 days) and two postneonatal periods. All odds ratios are relative to the reference temperature of 20°C, and are adjusted for mean relative humidity. The odds ratios are presented both on the original and the logarithmic scale.

P-Value for spline-modelled effect of maximum temperature:

	# of SIDS cases	Previous day	Same day
all ages (0-364 days)	187	0.97	0.74
1-2 months (28-89 days)	74	0.60	0.82
3-11 months (90-364 days)	96	0.67	0.17

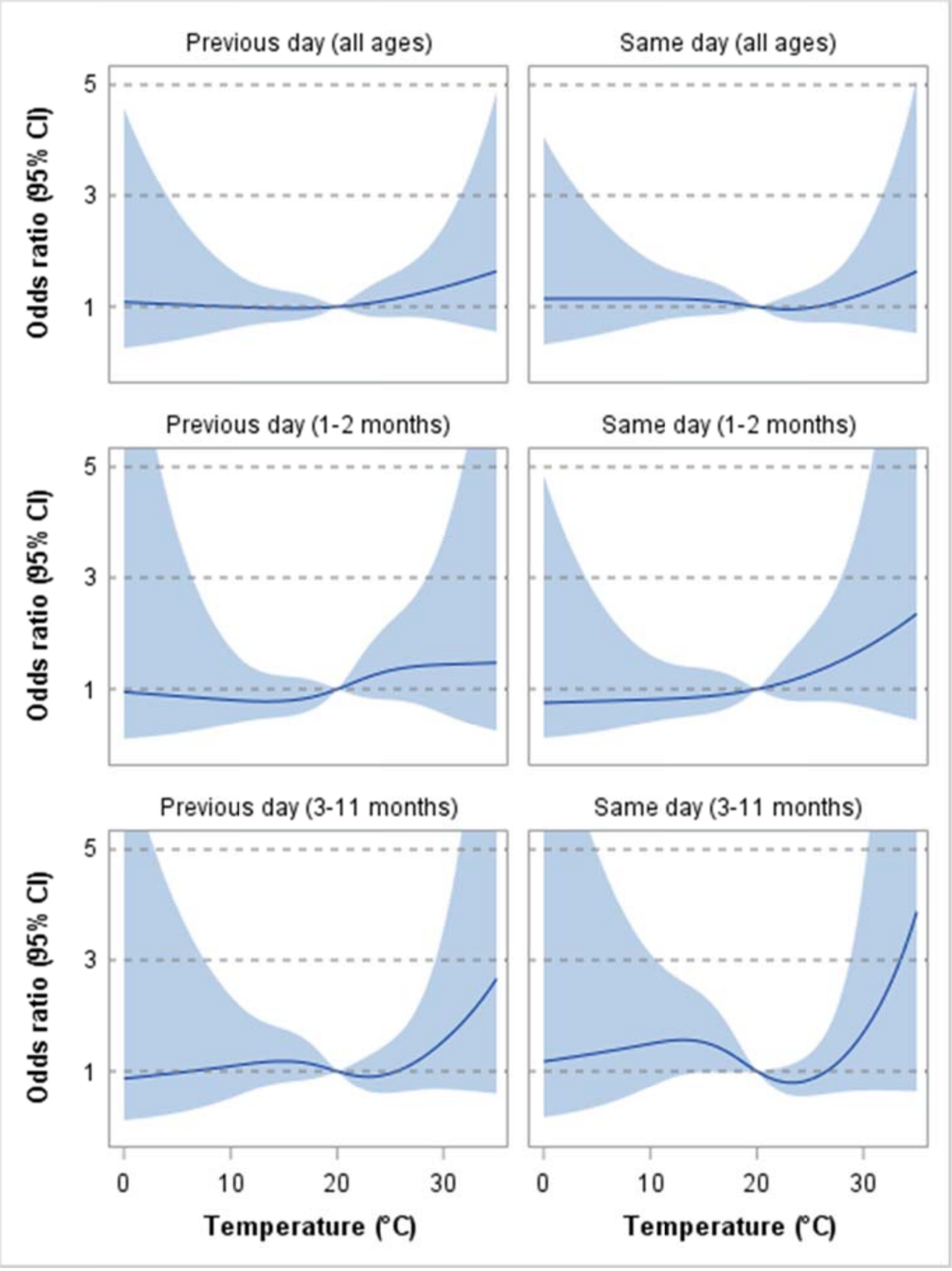
Previous day (all ages)
Same day (all ages)
Previous day (1-2 months)
Same day (1-2 months)
Previous day (3-11 months)
Same day (3-11 months)
Odds ratio (95% CI)
5
3
1
0
10
20
30
Temperature (°C)

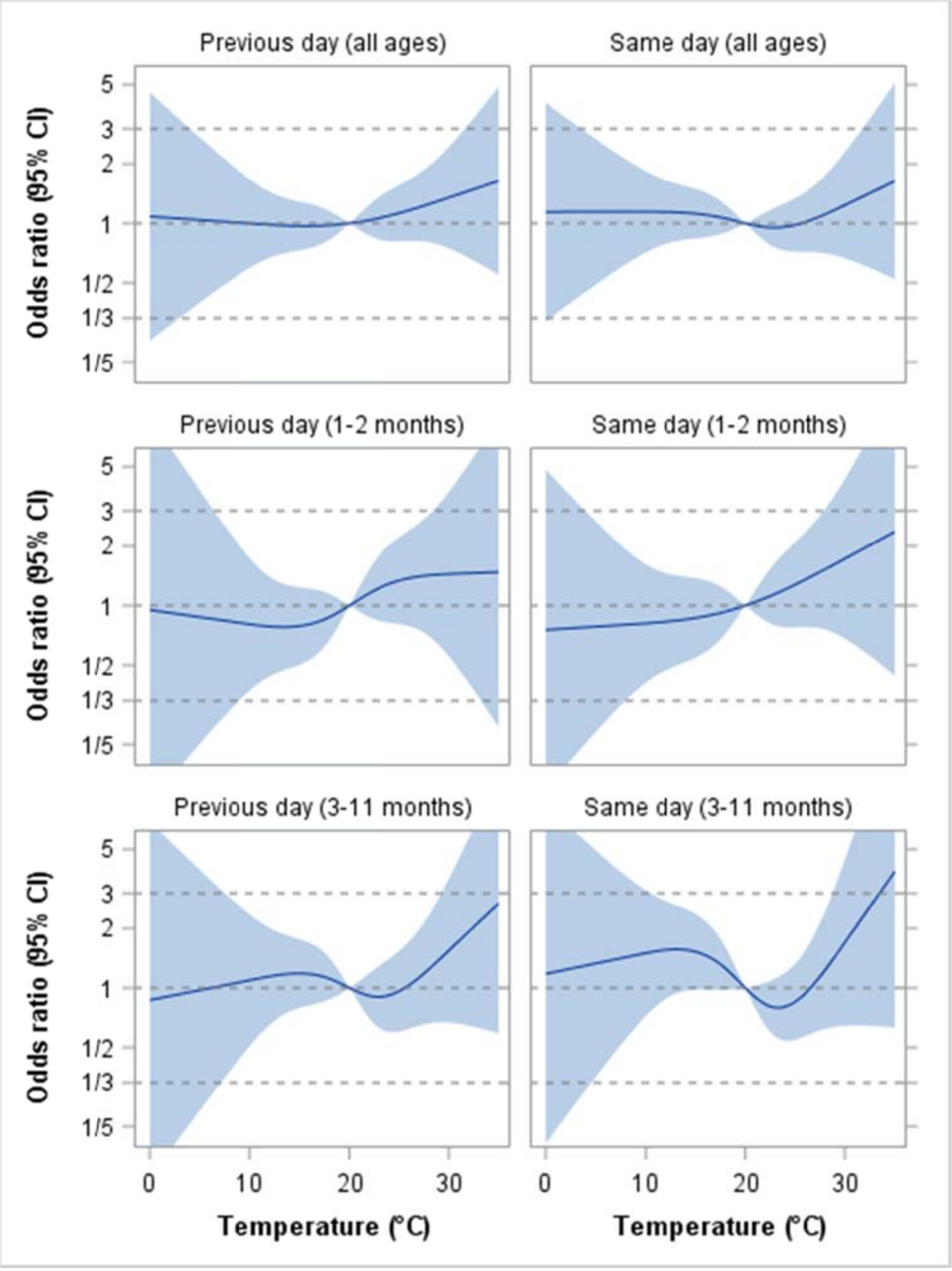
Previous day (all ages)
Same day (all ages)
Previous day (1-2 months)
Same day (1-2 months)
Previous day (3-11 months)
Same day (3-11 months)
Odds ratio (95% CI)
5
3
2
1
1/2
1/3
1/5
0
10
20
30
Temperature (°C)

Scenario 4: Association between maximum outdoor temperature and SIDS cases with mention of autopsy, Vienna, April–October 1984–2014. Maximum temperature was flexibly modelled with natural cubic splines with knots placed at the **5th, 27.5th, 50th, 72.5th and 95th percentiles**. For the day before and the day of the SIDS event, odds ratios (solid blue line) and 95% CI's (transparent blue area) are shown for all ages (0-364 days) and two postneonatal periods. All odds ratios are relative to the reference temperature of 20°C, and are adjusted for mean relative humidity. The odds ratios are presented both on the original and the logarithmic scale.

P-Value for spline-modelled effect of maximum temperature:

	# of SIDS cases	Previous day	Same day
all ages (0-364 days)	187	0.27	0.027*
1-2 months (28-89 days)	74	0.71	0.69
3-11 months (90-364 days)	96	0.48	0.056

*The overall model (which also includes mean relative humidity as covariate) was not statistically significant (p=0.17) when applying the two-sided significance level $\alpha = 0.05$.

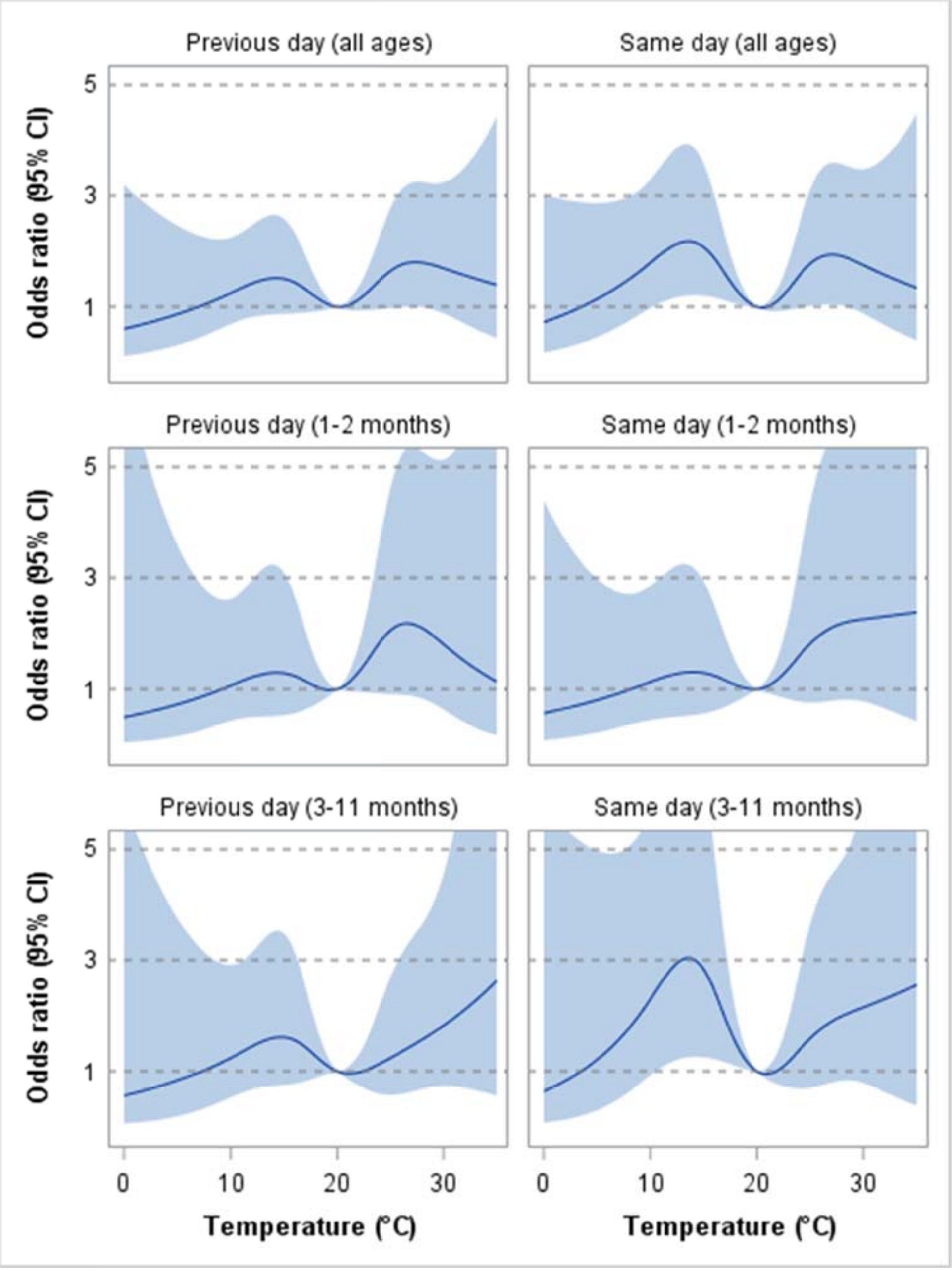
Previous day (all ages)
Same day (all ages)
Previous day (1-2 months)
Same day (1-2 months)
Previous day (3-11 months)
Same day (3-11 months)
Odds ratio (95% CI)
1
3
5
0
10
20
30
Temperature (°C)

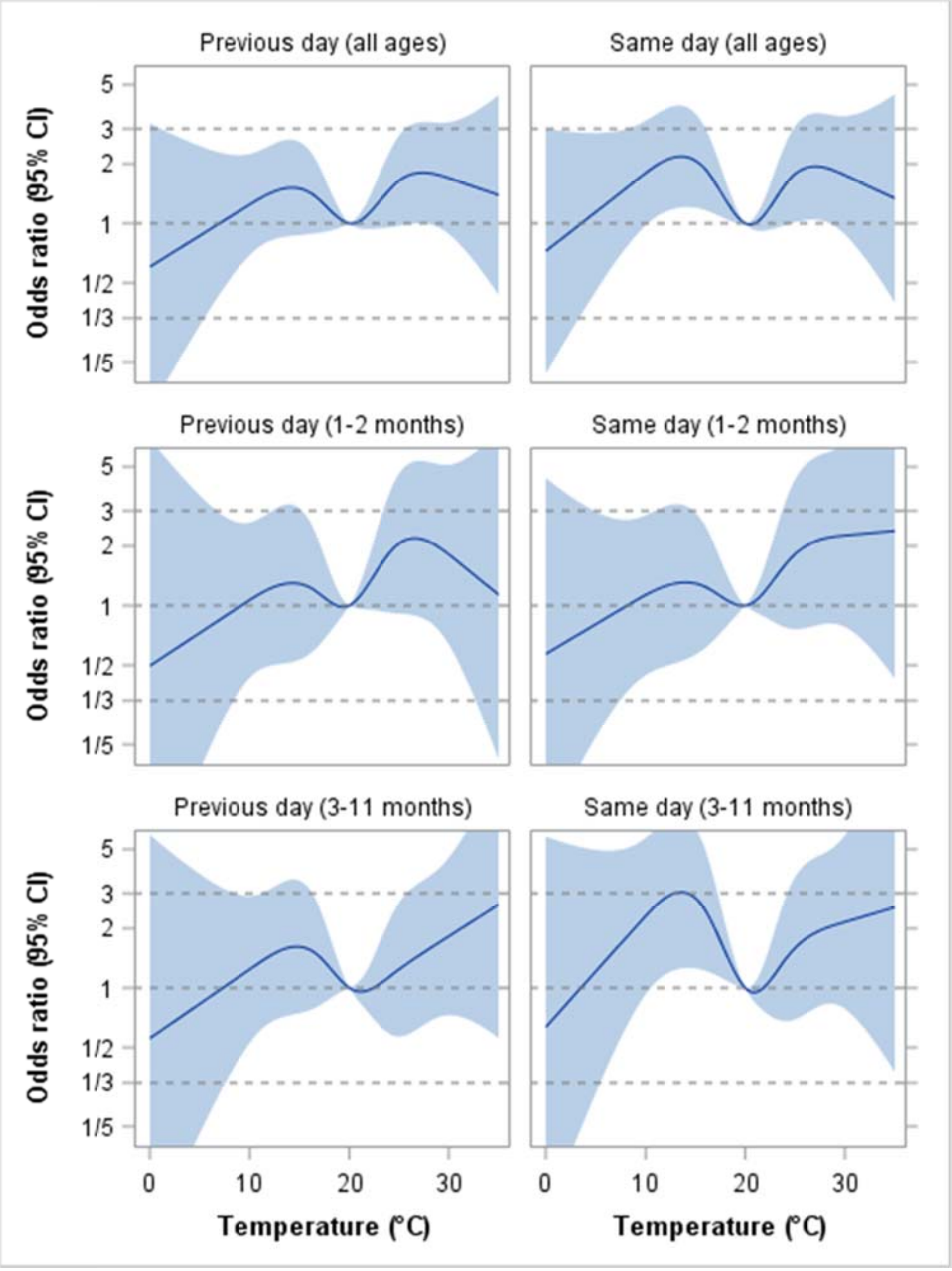
Previous day (all ages)
Same day (all ages)
Previous day (1-2 months)
Same day (1-2 months)
Previous day (3-11 months)
Same day (3-11 months)
Odds ratio (95% CI)
5
3
2
1
1/2
1/3
1/5
0
10
20
30
Temperature (°C)

Scenario 5: Association between maximum outdoor temperature and SIDS cases with mention of autopsy, Vienna, April–October 1984–2014. Maximum temperature was flexibly modelled with natural cubic splines with knots placed at the 10th, 50th, and 90th percentiles. For the day before and the day of the SIDS event, odds ratios (solid blue line) and 95% CI's (transparent blue area) are shown for all ages (0-364 days) and two postneonatal periods. All odds ratios are relative to the reference temperature of 20°C, **and are not adjusted for mean relative humidity**. The odds ratios are presented both on the original and the logarithmic scale.

P-Value for spline-modelled effect of maximum temperature:

	# of SIDS cases	Previous day	Same day
all ages (0-364 days)	187	0.55	0.67
1-2 months (28-89 days)	74	0.60	0.50
3-11 months (90-364 days)	96	0.53	0.51

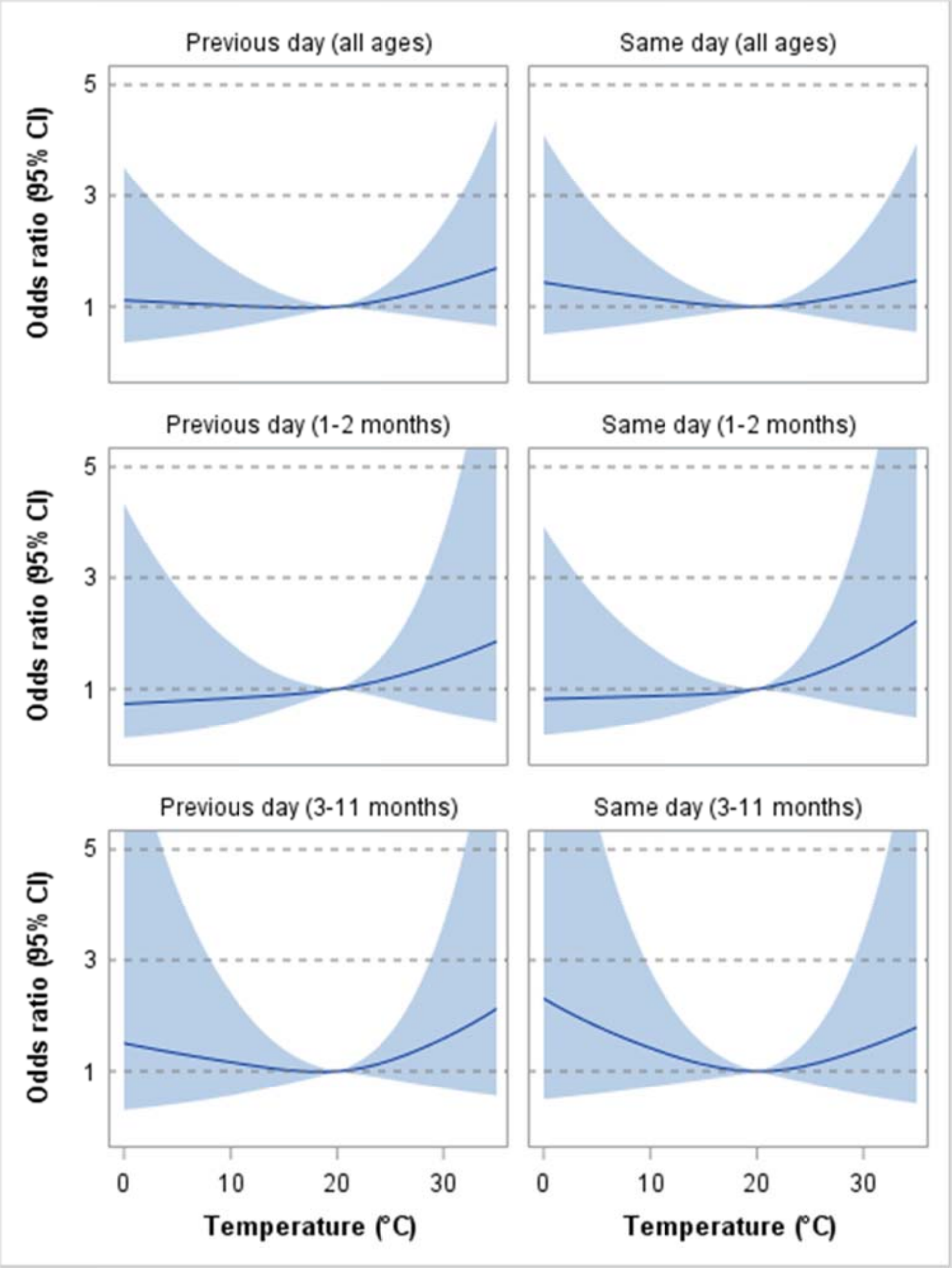
Previous day (all ages)
Same day (all ages)
Previous day (1-2 months)
Same day (1-2 months)
Previous day (3-11 months)
Same day (3-11 months)
Odds ratio (95% CI)
5
3
1
0
10
20
30
Temperature (°C)

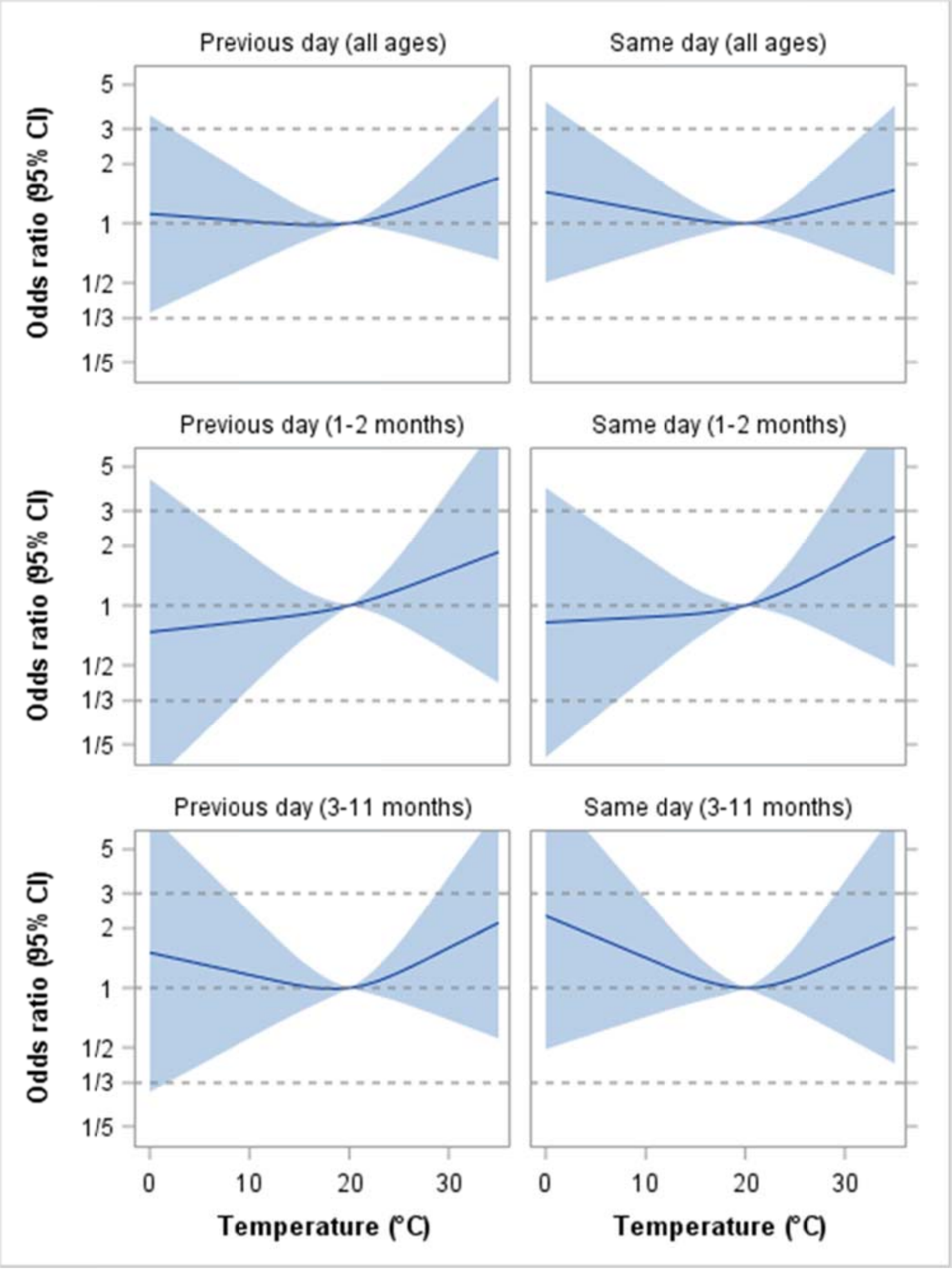

Previous day (all ages)
Same day (all ages)
Previous day (1-2 months)
Same day (1-2 months)
Previous day (3-11 months)
Same day (3-11 months)
Odds ratio (95% CI)
5
3
2
1
1/2
1/3
1/5
0
10
20
30
Temperature (°C)

Scenario 6: Association between maximum outdoor temperature and SIDS cases with mention of autopsy, Vienna, **June–August** 1984–2014. Maximum temperature was flexibly modelled with natural cubic splines with knots placed at the 10th, 50th, and 90th percentiles. For the day before and the day of the SIDS event, odds ratios (solid blue line) and 95% CI's (transparent blue area) are shown for all ages (0-364 days) and two postneonatal periods. All odds ratios are relative to the reference temperature of 20°C, and are adjusted for mean relative humidity. The odds ratios are presented both on the original and the logarithmic scale.

P-Value for spline-modelled effect of maximum temperature:

	# of SIDS cases	Previous day	Same day
all ages (0-364 days)	67	0.24	0.42
1-2 months (28-89 days)	24	0.27	0.33
3-11 months (90-364 days)	36	0.30	0.45

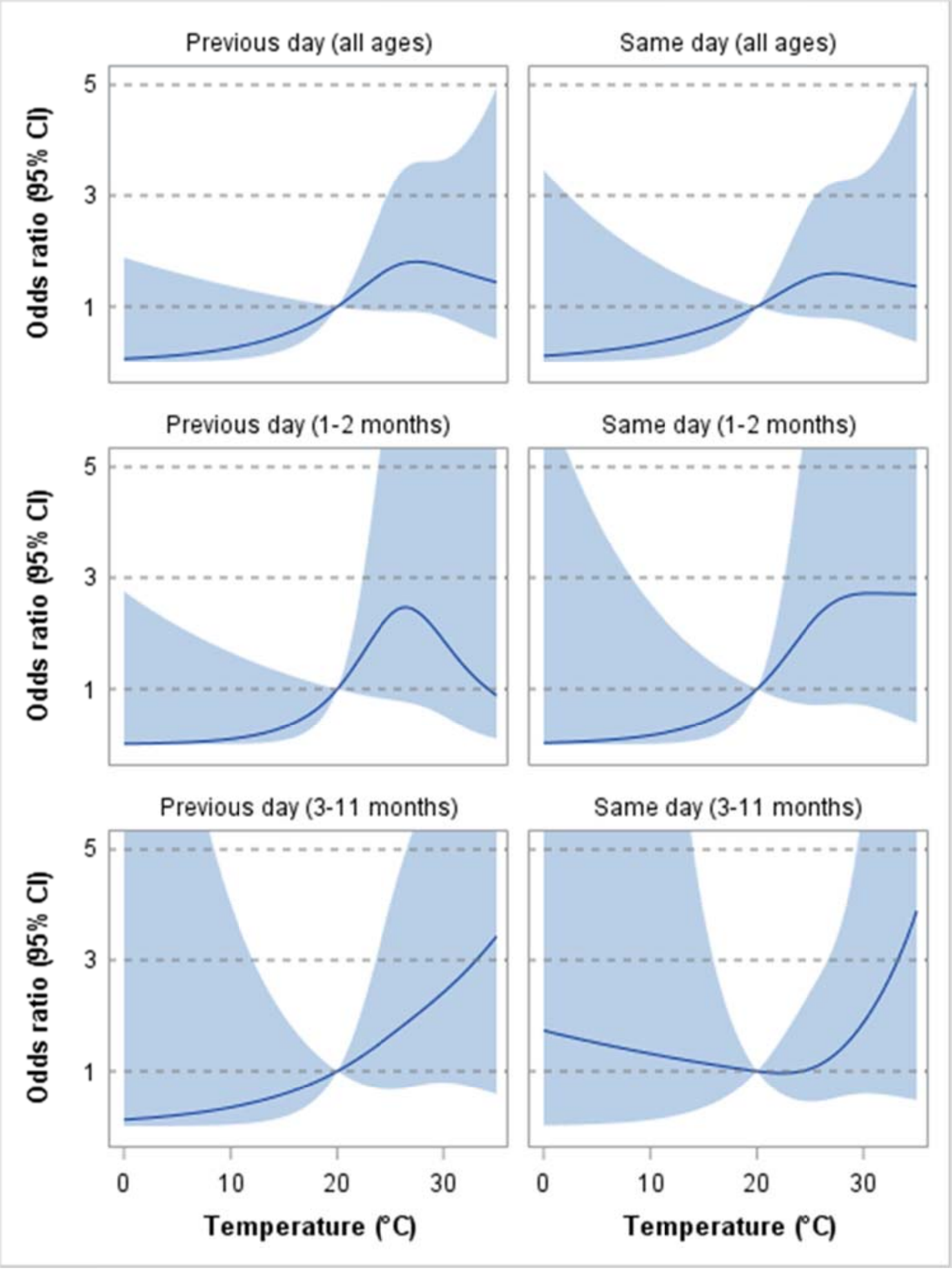
Previous day (all ages)
Same day (all ages)
Previous day (1-2 months)
Same day (1-2 months)
Previous day (3-11 months)
Same day (3-11 months)
Odds ratio (95% CI)
5
3
1
0
10
20
30
Temperature (°C)

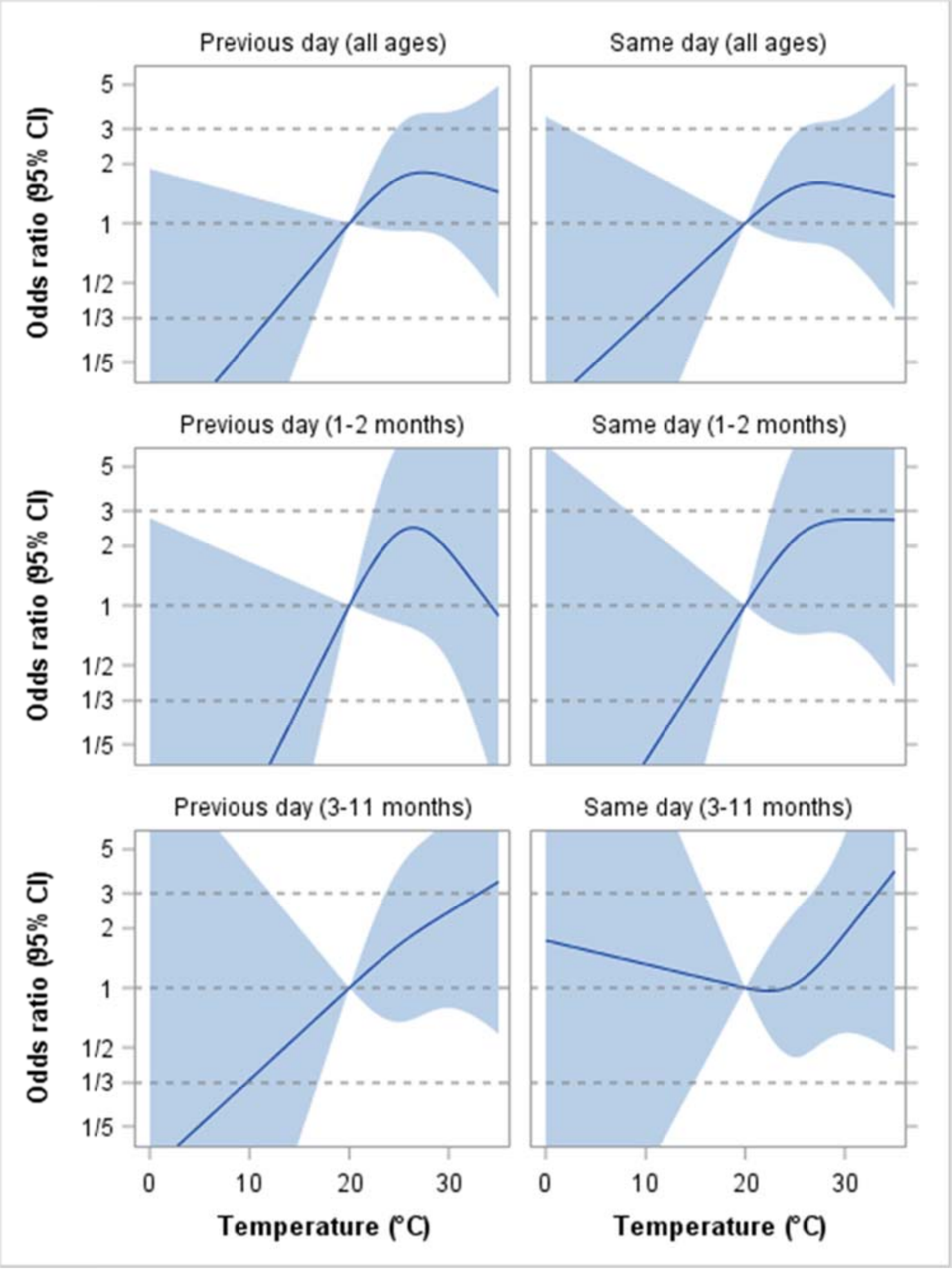

Previous day (all ages)
Same day (all ages)
Previous day (1-2 months)
Same day (1-2 months)
Previous day (3-11 months)
Same day (3-11 months)
Odds ratio (95% CI)
5
3
2
1
1/2
1/3
1/5
0
10
20
30
Temperature (°C)

Scenario 7: Association between maximum outdoor temperature and SIDS cases **(both with and without mention of autopsy)**, Vienna, April–October 1984–2014. Maximum temperature was flexibly modelled with natural cubic splines with knots placed at the 10th, 50th, and 90th percentiles. For the day before and the day of the SIDS event, odds ratios (solid blue line) and 95% CI's (transparent blue area) are shown for all ages (0-364 days) and two postneonatal periods. All odds ratios are relative to the reference temperature of 20°C, and are adjusted for mean relative humidity. The odds ratios are presented both on the original and the logarithmic scale.

P-Value for spline-modelled effect of maximum temperature:

	# of SIDS cases	Previous day	Same day
all ages (0-364 days)	201	0.71	0.89
1-2 months (28-89 days)	77	0.52	0.44
3-11 months (90-364 days)	103	0.69	0.49

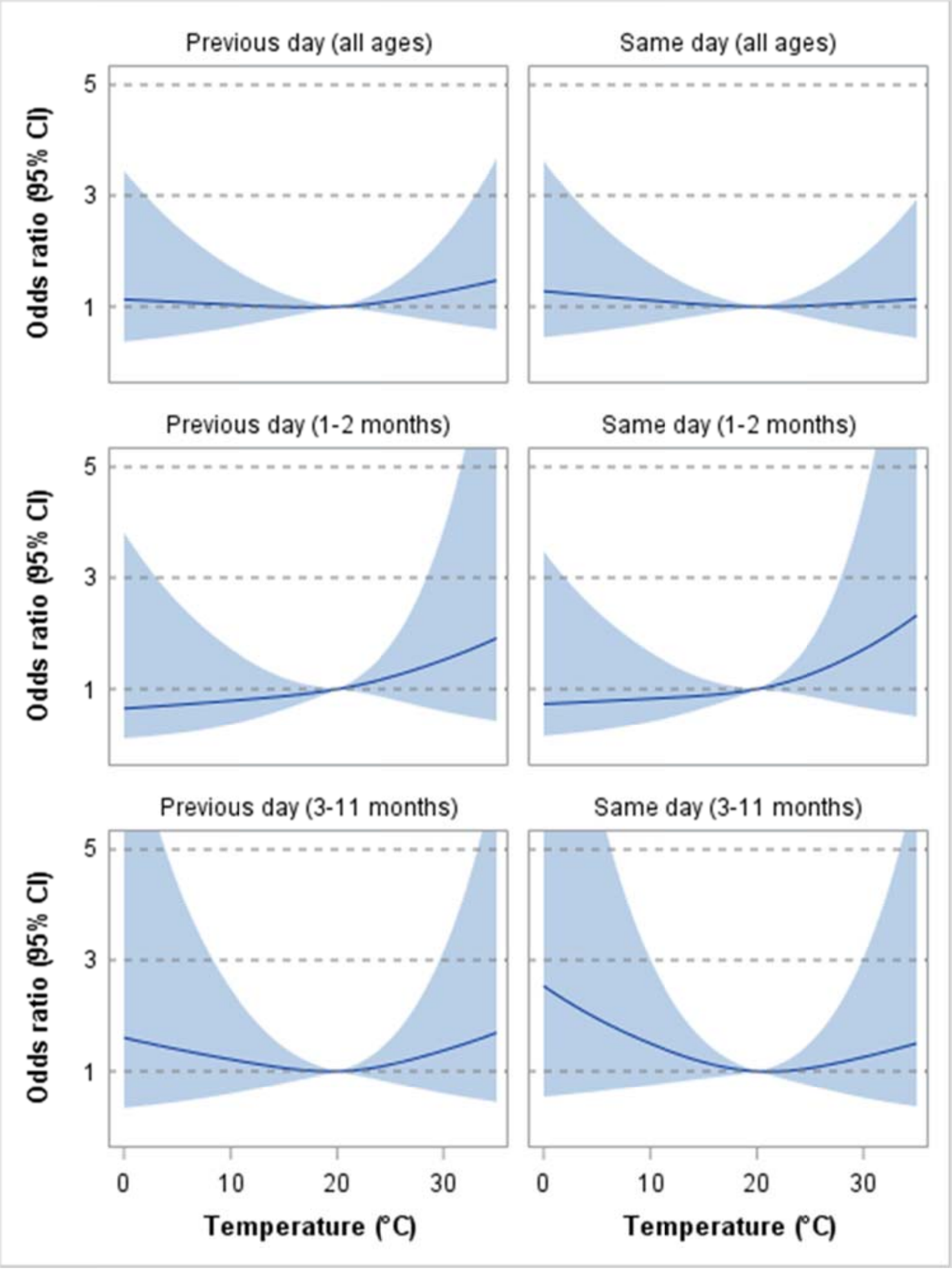
Previous day (all ages)
Same day (all ages)
Previous day (1-2 months)
Same day (1-2 months)
Previous day (3-11 months)
Same day (3-11 months)
Odds ratio (95% CI)
5
3
1
0
10
20
30
Temperature (°C)

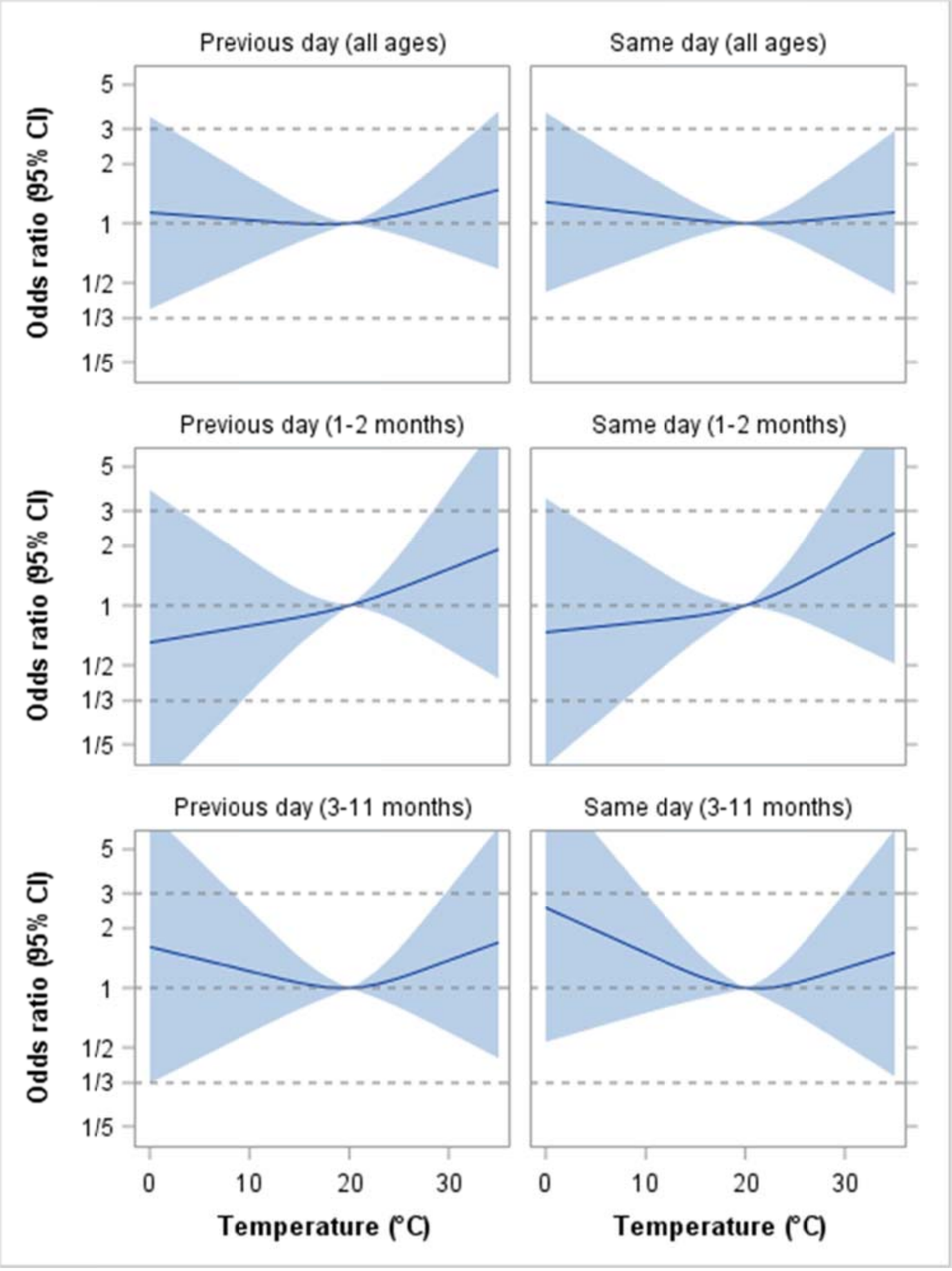
Previous day (all ages)
Same day (all ages)
Previous day (1-2 months)
Same day (1-2 months)
Previous day (3-11 months)
Same day (3-11 months)
Odds ratio (95% CI)
5
3
2
1
1/2
1/3
1/5
0
10
20
30
Temperature (°C)

Scenario 8: Association between maximum outdoor temperature and SIDS cases with mention of autopsy, **Vienna plus other Austrian cities (Klagenfurt, Linz, Wels, Salzburg, Graz, Innsbruck, Bregenz, Dornbirn)**, April–October 1984–2014. Maximum temperature was flexibly modelled with natural cubic splines with knots placed at the 10th, 50th, and 90th percentiles. For the day before and the day of the SIDS event, odds ratios (solid blue line) and 95% CI's (transparent blue area) are shown for all ages (0-364 days) and two postneonatal periods. All odds ratios are relative to the reference temperature of 20°C, and are adjusted for mean relative humidity. The odds ratios are presented both on the original and the logarithmic scale.

P-Value for spline-modelled effect of maximum temperature:

	# of SIDS cases	Previous day	Same day
all ages (0-364 days)	276	0.54	0.63
1-2 months (28-89 days)	110	0.84	0.23
3-11 months (90-364 days)	144	0.36	0.39

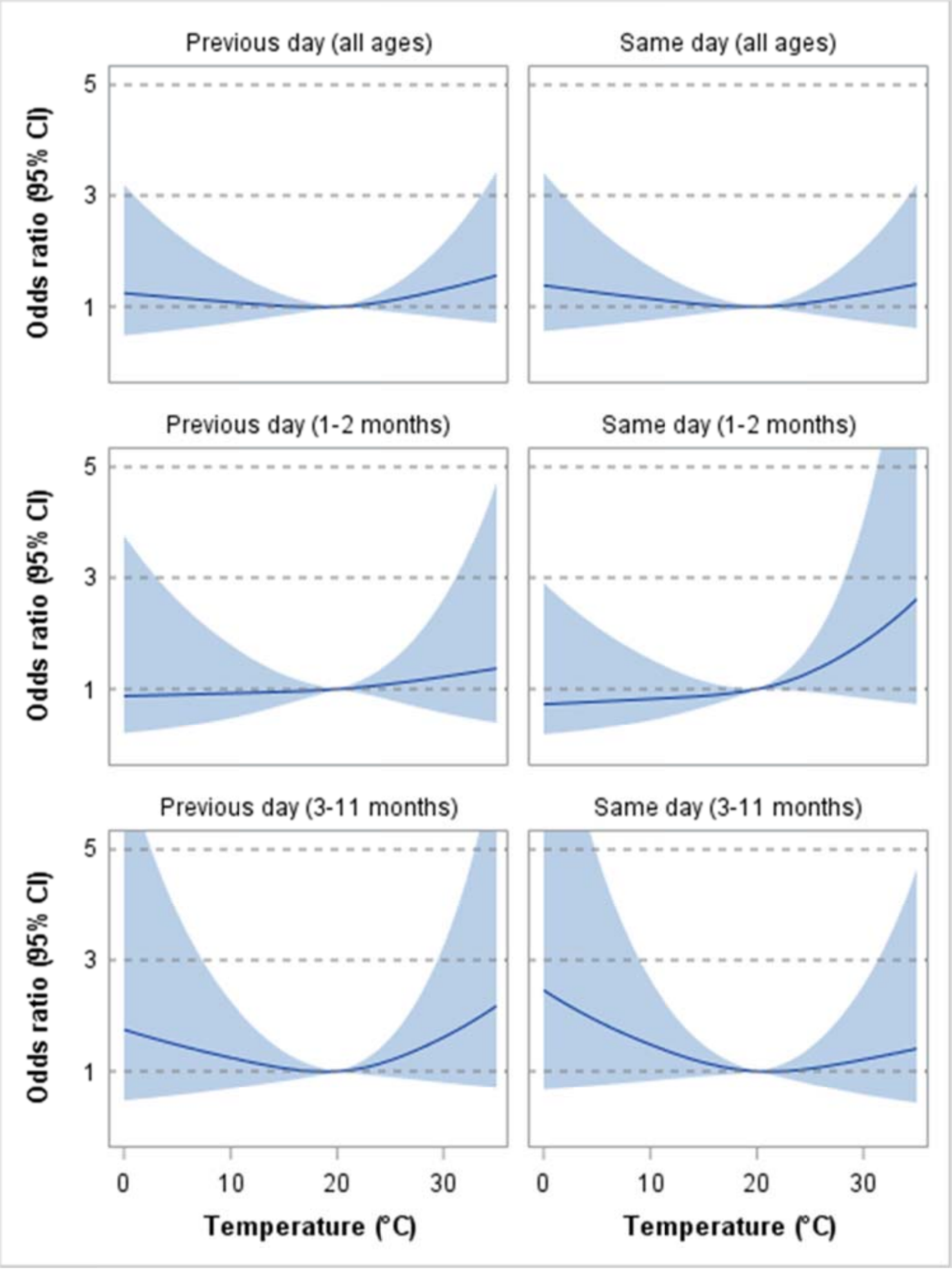
Previous day (all ages)
Same day (all ages)
Previous day (1-2 months)
Same day (1-2 months)
Previous day (3-11 months)
Same day (3-11 months)
Odds ratio (95% CI)
5
3
1
0
10
20
30
Temperature (°C)

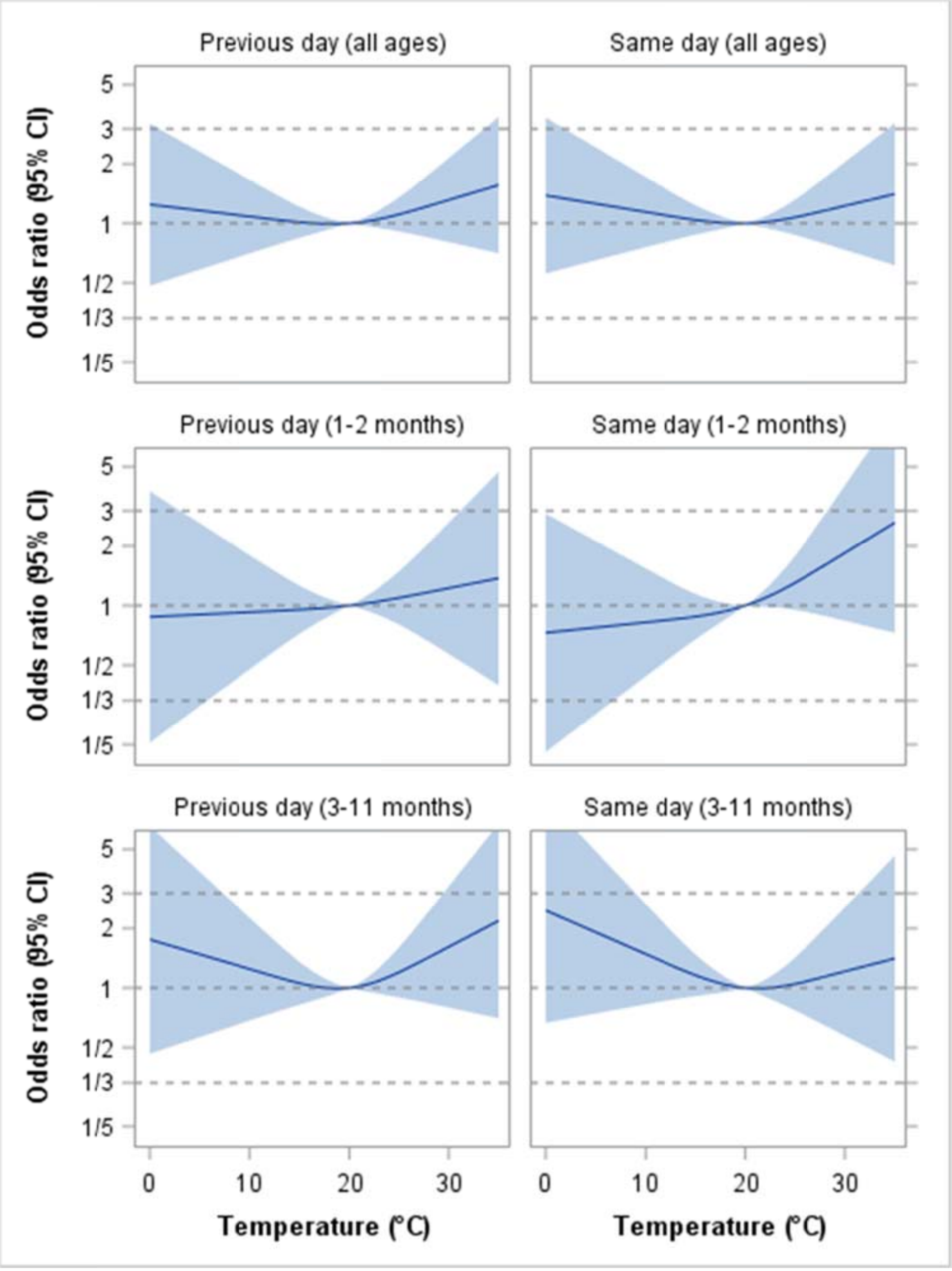
Previous day (all ages)
Same day (all ages)
Previous day (1-2 months)
Same day (1-2 months)
Previous day (3-11 months)
Same day (3-11 months)
Odds ratio (95% CI)
5
3
2
1
1/2
1/3
1/5
0
10
20
30
Temperature (°C)

Scenario 9: Association between maximum outdoor temperature and SIDS cases with mention of autopsy, Vienna, April–October 1984–2014. Maximum temperature was flexibly modelled with natural cubic splines with knots placed at the 10th, 50th, and 90th percentiles. For the day before and the day of the SIDS event, odds ratios (solid blue line) and 95% CI's (transparent blue area) are shown **for 3-6 months (90-212 days)**. All odds ratios are relative to the reference temperature of 20°C, and are adjusted for mean relative humidity. The odds ratios are presented both on the original and the logarithmic scale.

P-Value for spline-modelled effect of maximum temperature:

	# of SIDS cases	Previous day	Same day
3-6 months (90-212 days)	75	0.60	0.63

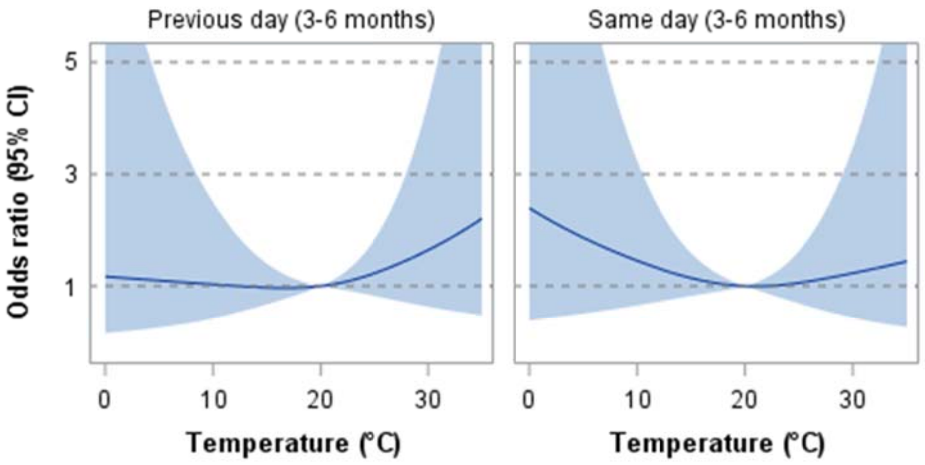

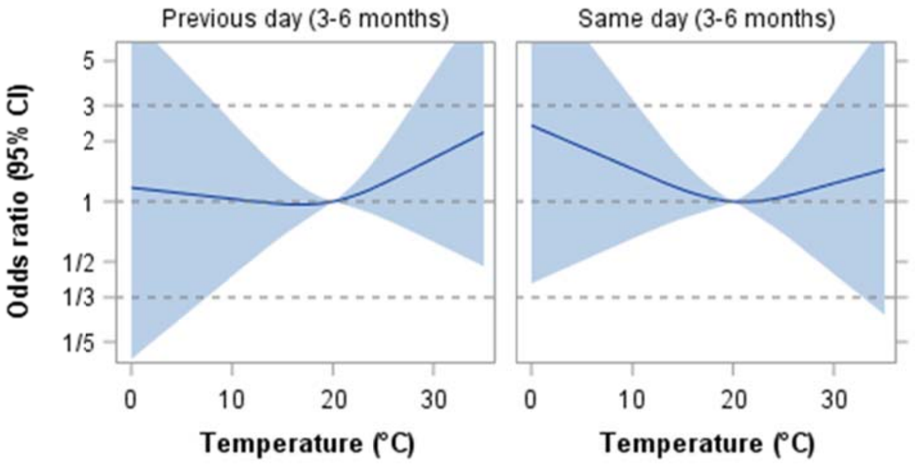

Scenario 10: Association between maximum outdoor temperature and SIDS cases with mention of autopsy, Vienna, April–October 1984–2014. Maximum temperature was flexibly modelled with natural cubic splines with knots placed at the 10th, 50th, and 90th percentiles. **For the second day before the SIDS event**, odds ratios (solid blue line) and 95% CI's (transparent blue area) are shown for all ages (0-364 days) and two postneonatal periods. All odds ratios are relative to the reference temperature of 20°C, and are adjusted for mean relative humidity. The odds ratios are presented both on the original and the logarithmic scale.

P-Value for spline-modelled effect of maximum temperature:

	# of SIDS cases	Second day before death
all ages (0-364 days)	187	0.25
1-2 months (28-89 days)	74	0.20
3-11 months (90-364 days)	96	0.51

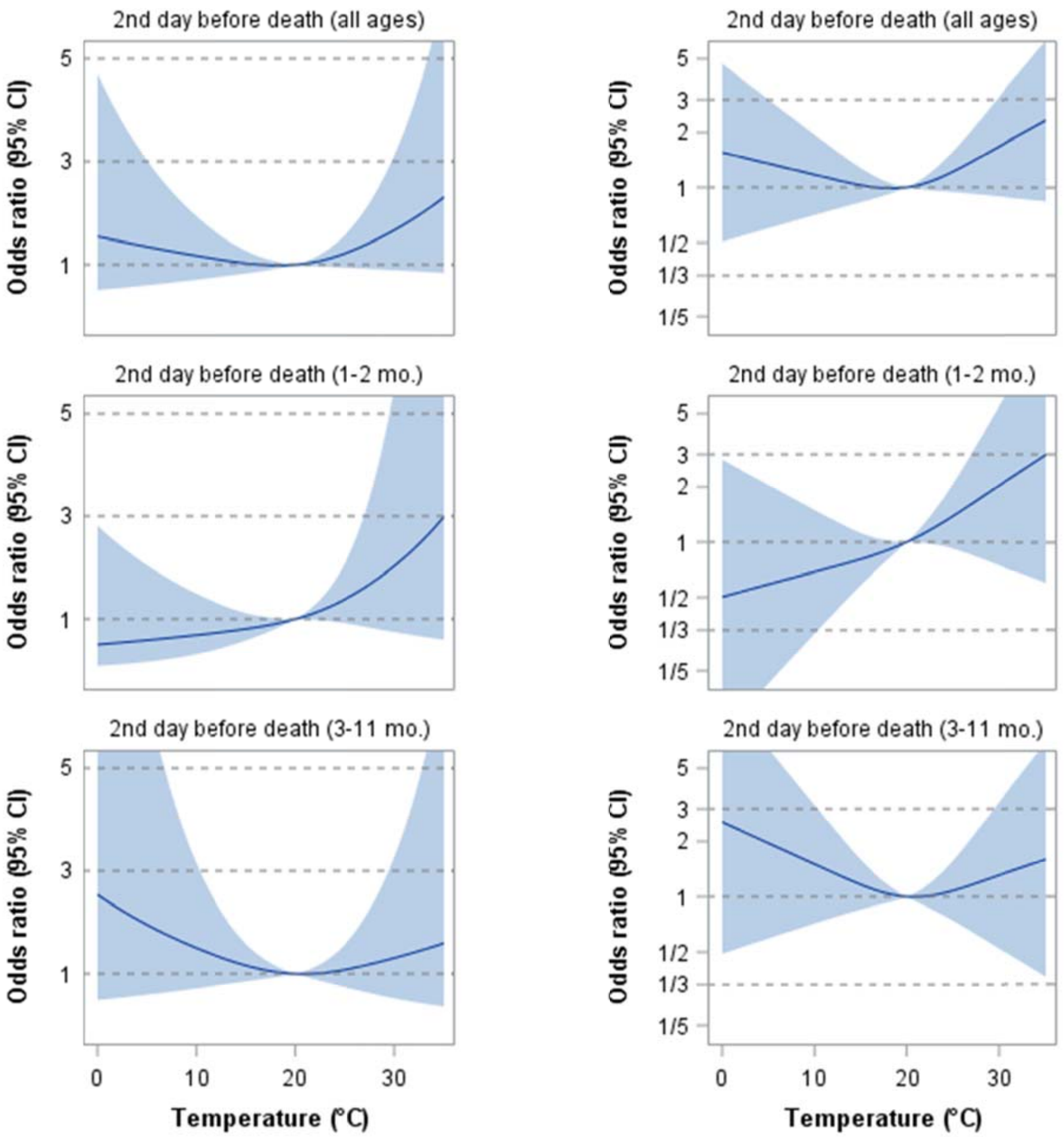
2nd day before death (all ages)
2nd day before death (1-2 mo.)
2nd day before death (3-11 mo.)
Odds ratio (95% CI)
5
3
2
1
1/2
1/3
1/5
0
10
20
30
Temperature (°C)
